# Netrin-1 inducing antiapoptotic effect of acute myeloid leukemia cells in a concentration-dependent manner through the Unc-5 netrin receptor B-focal adhesion kinase axis

**DOI:** 10.1080/15384047.2023.2200705

**Published:** 2023-04-10

**Authors:** Kainan Zhang, Xizhou An, Yao Zhu, Lan Huang, Xinyuan Yao, Xing Zeng, Shaoyan Liang, Jie Yu

**Affiliations:** a Department of Hematology and Oncology, Children’s Hospital of Chongqing Medical University; National Clinical Research Center for Child Health and Disorders; Ministry of Education Key Laboratory of Child Development and Disorders; Chongqing Key Laboratory of Pediatrics, Chongqing, China; b Pediatric research institute, Children’s Hospital of Chongqing Medical University, Chongqing, China

**Keywords:** Netrin-1, acute myeloid leukemia, UNC5B, FAK-Akt, apoptosis

## Abstract

Acute myeloid leukemia (AML) is a hematological malignancy that commonly occurs in children. The prognosis of pediatric AML is relatively poor, thus threatening the patient’s survival. The aberrant expression of the axon guidance factor, netrin-1, is observed in various types of malignancies, and it participates in the proliferation and apoptosis of tumor cells. Herein, we aimed to explore the role of netrin-1 in AML cells. Netrin-1 is highly expressed in AML patients. Proliferation and anti-apoptosis were observed in AML cells treated with netrin-1. The interaction between netrin-1 and Unc-5 netrin receptor B (UNC5B) was detected through coimmunoprecipitation, and UNC5B ribonucleic acid interference restrained the influence of netrin-1 on the AML cells. The phosphorylation of focal adhesion kinase-protein kinase B (FAK-Akt) was upregulated in AML cells treated with netrin-1. Both FAK and Akt inhibitors abrogated the effects of netrin-1 on the proliferation and apoptosis of AML cells. In conclusion, netrin-1 could promote the growth and reduce the apoptosis of AML cells in a concentration-dependent manner, and that these effects were mediated by activating the FAK-Akt signaling pathway via the UNC5B.

## Introduction

Acute myeloid leukemia (AML) is a life-threatening malignant disease that commonly occurs in children.^1^ It is a disorder of hematopoietic stem cells caused by chromosomal abnormalities or gene mutations that lead to the overproduction of neoplastic clonal myeloid stem cells.^2^ AML accounts for approximately 15–20% of pediatric leukemia cases.^3^ In recent decades, the overall survival (OS) rate of children with AML has remarkably increased due to the intensification of chemotherapy, allogeneic hematopoietic stem cell transplantation, and improvement in supportive care.^4^ However, the outcomes of different biological subtypes of AML vary.^5^ Moreover, primary refractory or relapsed pediatric AML, which accounts for 35–48% of pediatric AML cases in 5 years,^6–8^ results in significant morbidity and mortality, and the long-term survival in patients with these features remains poor.^9^ Therefore, the exploration and development of novel treatments for children in high-risk groups with poor outcomes are urgently required.

Resistance to apoptosis is one of the features of tumor cells,^10^ which contributes to carcinogenesis, tumor progression, and treatment resistance.^11^ In AML cells, resistance to apoptosis is a significant feature that can induce relapse of the primary disease. B-cell lymphoma 2 (Bcl-2), an apoptosis suppressor gene, is highly expressed in the bone marrow of AML patients.^12^ The overexpression of Bcl-2 induces resistance to chemotherapy drugs in AML cells,^13^ thus leading to poor prognosis, such as relapse in AML.^14^

Netrin-1, which serves as an axon guidance factor during nervous system development,^15^ can induce antiapoptotic effects when it binds to its receptors by inhibiting caspase cleavage.^16^ Deleted in Colorectal Carcinoma(DCC) and UNC5 homologs, which are also receptors of netrin-1, regulate the proliferation and apoptosis of tumor cells by activating various downstream pathways.^17,18^ Netrin-1 could be upregulated in pancreatic ductal adenocarcinoma and confers resistance to apoptosis in tumor and endothelial cells in vitro.^19^ A previous study by our group suggested that netrin-1 could induce antiapoptotic effects in B-cell acute lymphoblastic leukemia (B-ALL) cells, another type of hematopoietic malignancy.^20^ Netrin-1 induces antiapoptotic effects by binding to dependent receptors and their downstream signaling pathways, such as mitogen-activated protein kinase (MAPK)^20–22^ and phosphoinositide 3-kinase-protein kinase B (PI3K-Akt).^23^ However, the mechanism by which the signal transduction pathway mediates the netrin-1-dependent antiapoptotic effect on malignant cells is not thoroughly understood. Whether netrin-1 can induce antiapoptotic effects in other hematopoietic malignancies, such as AML, and the signal transduction pathway through which netrin-1 could exert such action have seldom been investigated.

In the present study, we aimed to investigate the role netrin-1 played in AML cells and its underlying mechanisms. First, the serum level of netrin-1 in clinical samples were measured to compare the expression level of netrin-1 between patients and children in the control group. Recombinant netrin-1 was then used to treat AML cells to explore its effect on apoptosis. Coimmunoprecipitation (co-IP) and receptor gene ribonucleic acid interference (RNAi) were used to explore and verify the receptor binding to netrin-1 in AML cells. Finally, western blotting and signal transduction pathway inhibitor treatment were used to explore and verify the signal transduction pathway downstream of netrin-1.

## Results

### Serum netrin-1 level in AML and control patients

**Figure 1.**
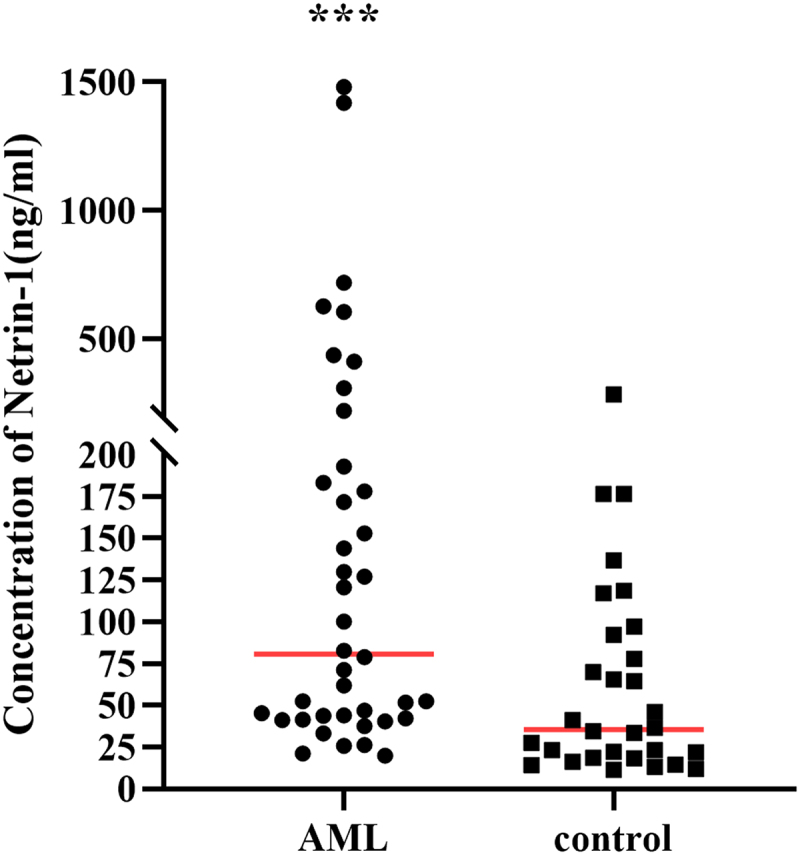
Serum Netrin-1 level in AML patients and control patients.

The serum netrin-1 expression level in patients with AML and nonhematological malignant diseases (immune thrombocytopenic purpura, IPT; iron-deficiency anemia, IDA, etc., control group) were detected using enzyme-linked immunosorbent assay (ELISA) (Figure 1). The clinical information of all patients was collected to analyze the relationship between serum netrin-1 expression level and clinical indications. The netrin-1 expression level in the AML group was significantly higher than that in the control group (*P* < .001). However, the serum netrin-1 expression levels were significantly lower in the high-risk (HR) than that in low- or intermediate-risk (LR + IR) AML patients at first diagnosis (*P* < .001; Table 1). These results suggest that the mechanism underlying the contribution of netrin-1 to the development of AML is complex.

**Table 1.** Relevance between serum Netrin-1 level and clinical indications of AML patients.

Clinical Indications	n	Netrin-1(ng/ml)	*P*
Sex			0.5655
Male	26	66.5(25.7–1420.0)	
Female	14	141.5(19.9–1481.0)	
Age			0.7427
＞10 years	11	120.6(40.7–1481.0)	
≤10 years	29	71.1(19.9–1420.0)	
FAB Classification			0.4487
M1	1	43.7	
M2	16	80.8(19.9–718.6)	
M3	13	52.5(21.1–1481.0)	
M4	1	128.9	
M5	5	45.3(33.2–143.9)	
M7	4	298.1(71.1–1420.0)	
WBC			0.9560
＞20 × 10^9^/L	15	78.9(25.7–1481.0)	
≤20 × 10^9^/L	25	82.6(19.9–1420.0)	
PLT			0.3225
＞20 × 10^9^/L	19	82.6(33.2–718.6)	
≤20 × 10^9^/L	11	46.9(19.9–1481.0)	
Hb			0.7539
＞90 g/L	10	135.9(19.9–627.0)	
≤90 g/L	30	76.9(21.1–1481.0)	
CNSL (21 cases)			0.0346*
Yes	3	192.9(178.2–605.8)	
No	18	52.0(19.9–1481.0)	
Abnormal chromosome （27 cases）			0.1140
Yes	25	52.5(21.1–1581.0)	
No	2	199.2(178.2–220.2)	
LDH			0.5115
＞500IU	13	126.9(25.7–1420.0)	
≤500IU	27	62.0(19.9–1481.0)	
Risk Classification (Except M3, 22 cases)			＜0.0001****
LR+IR	11	178.2(62.0–1420.0)	
HR	11	45.3(26.4–82.6)	
D28 MRD (20 cases)			0.1680
Positive	5	51.6(41.2–78.9)	
Negative	15	120.6(37.5–627.0)	

*P＜0.05，****P＜0.0001.

### Netrin-1 regulating the growth and apoptosis of AML cells at different concentrations

**Figure 2.**
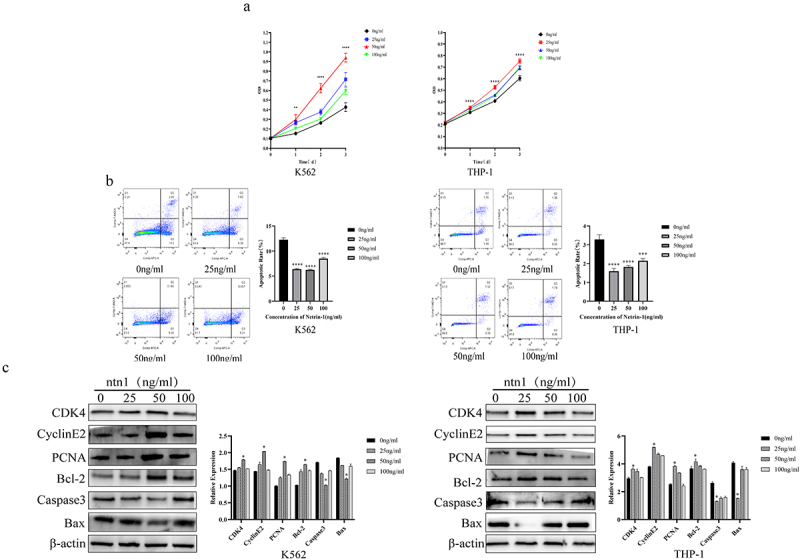
Regulation of Netrin-1 on the growth and apoptosis of AML cells.

The AML cell lines, K562 and THP-1, were treated with recombinant human netrin-1 at concentrations of 0, 25, 50, and 100 ng/mL, respectively. Cell Counting kit-8 (CCK-8) assays were performed to determine the cell growth curves. The growth curves of K562 and THP-1 cells treated with different concentrations of netrin-1 significantly increased compared with those of cells in the 0 ng/mL group (Figure 2a). Flow cytometry was used to detect the apoptotic rate of AML cells treated with different concentrations of netrin-1, and the apoptotic rate of both cell types was significantly decreased (Figure 2b) in the group with netrin-1 treatment. The expression of proliferation-related genes, *PCNA*, *CDK4*, and *cyclin E2*, and apoptosis-related genes, *bcl-2*, cleaved caspase-3, and Bax, was detected using western blotting after 24 h of treatment with different concentrations of netrin-1 (Figure 2c). Treatment with netrin-1 upregulated the expression of proliferative and antiapoptotic genes and downregulated the expression of proapoptotic genes. Overall, netrin-1 has the potential to promote proliferation and induce resistance to apoptosis in K562 and THP-1 cells.

### Netrin-1 regulating anti-apoptosis through the UNC5B in AML cells

**Figure 3.**
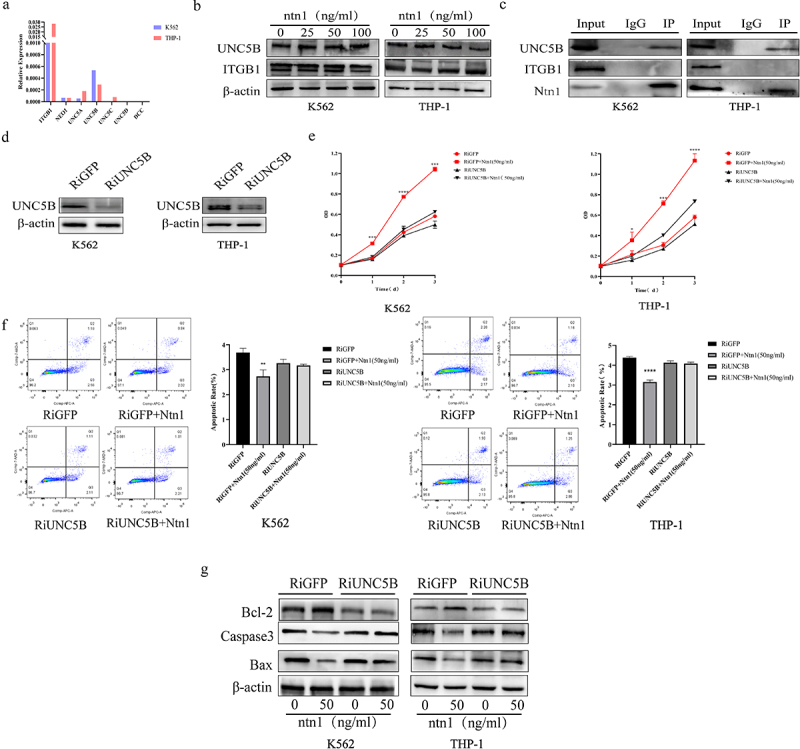
The receptor through which Netrin-1 regulate the AML apoptosis.

To investigate the receptor interaction with netrin-1, real-time reverse transcription-polymerase chain reaction (RT-PCR) (Figure 3a) and western blotting (Figure 3b) were performed to detect the expression of netrin-1 receptors. Integrin β1 (ITGB1) and Unc5 homolog B (UNC5B) were highly expressed in K562 and THP-1 cells. Changes in the netrin-1 level did not affect the receptor expression (Figure 3b). Since the optimum concentrations of netrin-1 for apoptosis regulation were 25 ng/mL and 50 ng/mL, 50 ng/mL of netrin-1 was chosen as the treatment concentration for subsequent experiments. Co-IP assays were performed to detect the possible receptor of netrin-1; UNC5B, but not integrin β1, interacted with netrin-1 at a concentration of 50 ng/mL (Figure 3c). Furthermore, the UNC5B expression interference was induced by infecting the K562 and THP-1 cells with UNC5B-specific RNAi lentivirus, and the expression interference was confirmed using western blotting (Figure 3d). CCK-8 and flow cytometry assays were performed to detect the proliferation and apoptotic rates of UNC5B RNAi AML cells and GFP RNAi control AML cells treated with either 0 ng/mL or 50 ng/mL netrin-1 for 24 h. Knockdown of UNC5B abolished the promotion of proliferation (Figure 3e) and the antiapoptotic effect (Figure 3f) of netrin-1. Western blotting showed that the differences in expression of apoptosis- and proliferation-related genes induced by netrin-1 treatment also disappeared in the UNC5B interfered cells (Figure 3g). In short, netrin-1 may promote the proliferation and inhibit the apoptosis of AML cells by binding to the receptor UNC5B.

### Netrin-1 regulating the anti-apoptosis by activating the Akt-NF-κB pathways in AML cells

**Figure 4.**
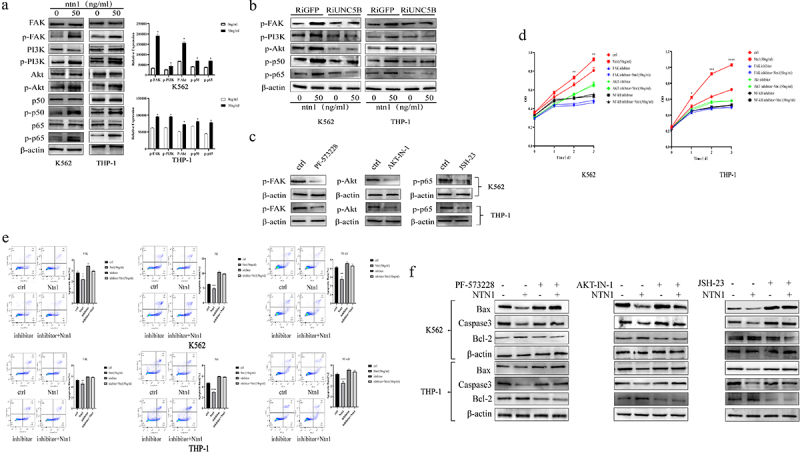
The downstream signal transduction pathway of Netrin-1.

Furthermore, we investigated the downstream signaling pathways through which netrin-1 regulates cell proliferation and apoptosis. Western blotting was performed to detect the phosphorylation level of the key signal transducers in the common pathways involved in the development of malignancy; the phosphorylation of FAK, PI3K, Akt, and Nuclear Factor- Kappa B (NF-κB) was significantly elevated in AML cells treated with 50 ng/mL netrin-1 for 6 h (Figure 4a). Interference with UNC5B expression in AML cells decreased the phosphorylation of FAK, PI3K, Akt, P50, and P65 (Figure 4b). Furthermore, the AML cells were treated with FAK (PF-573228, 20 nM, 24 h), Akt (AKT-IN-1, 5.21 μM, 24 h), and NF-κB inhibitors (JSH-23, 35.5 μM, 24 h)(Figure 4c). CCK-8 (Figure 4d) and flow cytometry assays (Figure 4e) were performed to detect the growth curve and apoptosis rate in normal and inhibitor-treated AML cells treated with either 0 ng/mL or 50 ng/mL netrin-1. These results suggested that these inhibitors could abolish the effect of netrin-1 on cell growth and apoptosis. Western blotting for proliferation- and apoptosis-related genes was performed; the FAK, Akt, and NF-κB inhibitors prevented the regulation of proliferation and apoptosis-related genes of netrin-1 (Figure 4f). In summary, netrin-1 activated the FAK-Akt-NF-κB signaling pathway via the UNC5B to induce proliferation and apoptosis in AML cells, while the pathway inhibitors reduced these effects.

## Discussion

Netrin-1 is a member of the laminin-like family of proteins. Netrin-1 is involved in the regulation of angiogenesis, inflammation, tissue remodeling, and cancers.^24^ The function of netrin-1 in cancers has been extensively studied. The upregulation of netrin-1 can be observed in many types of cancers, such as gastric cancer,^25^ ovarian cancer,^26^ and neuroblastoma,^27^ and it promotes their progression. By contrast, a reduction in netrin-1 level was observed in patients with prostate and brain cancers.^28,29^ Netrin-1 suppresses pancreatic cancer through the activation of the UNC5B receptor.^30^ These results indicate that the role of netrin-1 in cancer is variable, which might be due to its binding to different receptors or different origins of cancer cells. Our previous study found that netrin-1 interacts with UNC5B in B-ALL. However, the function of netrin-1 in AML and its effect on the progression of fatal diseases have not yet been studied. In this study, we used ELISA to detect the concentration of netrin-1 in the serum of AML patients and found that netrin-1 levels were increased. CCK-8, flow cytometry, and western blot analyses demonstrated that when 50 ng/mL netrin-1 was administered, the AML cells grew the fastest and died the least. Proliferative genes (CDK4, PCNA, and cyclin E2) and apoptosis-promoting genes (cleaved caspase-3 and Bax) were highest in the 50 ng/mL group, while the antiapoptotic gene (Bcl-2) was the lowest. In summary, netrin-1 promoted cell proliferation and apoptosis at specific concentrations. This function has rarely been reported in malignant solid tumors. Therefore, attention should be paid to patients whose serum netrin-1 expression is at this specific level. These patients may develop the disease faster and have worse prognosis.

The functions of netrin-1 are mostly mediated by receptors, including DCC, Unc5 homologs, integrins, and Neo1.^31–33^ The diversity of binding receptors makes netrin-1 a multifunctional molecule that plays different roles in specific cells depending on the receptors it bind with.^20,21,34,35^ In this study, we performed RT-PCR and western blotting and found that UNC5B was expressed in AML cells, whereas DCC was absent. Co-IP analysis showed that the UNC5B receptor bound with netrin-1 to induce antiapoptotic effects in AML cells. Further verification of the function of UNC5B as netrin-1 receptor was achieved because the RNAi of UNC5B expression in AML cells abrogated the antiapoptotic effect of netrin-1. CCK-8, flow cytometry, and western blot results showed that both proliferation and anti-apoptosis abilities were almost the same in UNC5B AML cells in the 0 ng/mL and 50 ng/mL netrin-1 groups. These results suggest that the UNC5B receptor is necessary for netrin-1 function in AML cells, which is consistent with the results of our previous study on B-ALL.^20^ Detection of the netrin-1 receptor is helpful for exploring the downstream pathway. Moreover, the necessity of UNC5B for netrin-1 may make it a potential target for future treatments.

Netrin-1 regulates cancer cell proliferation through the multiple signal transduction pathways. Yin et al.^36^ showed that netrin-1 participated in the proliferation and invasion of gastric cancer via the Neo1 through the Jak/Stat, PI3K/Akt, and Erk/MAPK pathways. Other studies indicated that netrin-1 could interact with receptors, DCC, or Unc5 family members and activate the PI3K-Akt pathway, which further activates NF-κB and leads to the transcriptional activation of antiapoptotic genes.^37–39^ FAK is a key transducer downstream of netrin-1 that promotes cell survival and anti-apoptosis by activating the kinase-dependent pathways, including the PI3K/Akt cascade.^40^ In the present study, the FAK-Akt-NF-κB signaling pathway was activated after treatment with netrin-1 detected by western blot assay and induced proliferation and anti-apoptosis effects in AML cells. In RNAi UNC5B cells, western blot results showed that netrin-1 did not activate the FAK-Akt pathway. The application of pathway inhibitors (FAK, Akt, and NF-κB) decreased the proliferation and anti-apoptosis effect of netrin-1 in AML cells, which were detected through CCK-8, flow cytometry, and western blotting. These results suggest that the UNC5B/FAK-Akt pathway is essential for the function of netrin-1 in AML cells. These findings are similar to those observed in other cancer cells, despite their different tissue origins. Targeting molecules involved in this pathway may delay disease progression and drug resistance.

In our study, we used a concentration ladder of 0, 25, 50, and 100 ng/mL of netrin-1 to treat AML cells. Compared with the 0 ng/mL group, all other groups showed a significant increase in proliferation and a decrease in apoptosis. However, netrin-1 was the most effective when used at concentrations of 25 ng/mL and 50 ng/mL, and the effect decreased in the 100 ng/mL group, thus suggesting that a bell-shaped, concentration-dependent manner was the potential paradigm of its function in AML cells. To date, only a few studies have investigated the bell-shaped concentration-dependent effect of netrin-1. Some studies have shown that other molecules participate in disease development in a bell-shaped concentration-dependent manner.^41,42^ However, the specific concentration-dependent mechanism underlying the role of netrin-1 in AML cells remains unknown and requires further investigation.

Taken together, our results indicated that netrin-1 plays an important role in the occurrence and development of AML. In addition, we identified the key mechanism for netrin-1 in the regulation of AML cell proliferation and apoptosis via the UNC5B through the FAK-Akt-NF-κB pathway. This may provide a new approach for treating AML by targeting the netrin-1/UNC5B/FAK-Akt signaling axis.

## Materials and methods

### Participants of this study

Peripheral blood serum was obtained from 40 patients newly diagnosed with AML who were admitted to the Department of Hematology and Oncology, Children’s Hospital of Chongqing Medical University between November, 2019 and October, 2020. All patients were diagnosed according to the 2016 revision of the World Health Organization classification of myeloid neoplasms and acute leukemia^5^. The treatment plan and risk stratification were based on the recommendations of an international expert panel.^43^ Simultaneously, the peripheral blood sera of 30 patients with nonhematological malignant diseases (ITP, IDA, etc.) (nonmalignant control group) were collected. This study was approved by the Ethics Committee of the Children’s Hospital of Chongqing Medical University (no. 2019–253), and informed consent was obtained from the parents or guardians of the children.

### Antibodies and reagents

Recombinant netrin-1 (#6419-N1-025) was purchased from R&D Systems Inc. (Minneapolis, MN, USA). The FAK inhibitor, PF-573228 (HY-10461); Akt inhibitor, AKT-IN-1 (HY-18296); and NF-κB inhibitor, JSH-23 (HY-13982); were purchased from MCE China (Shanghai, China). The antinetrin-1 antibody with a His-tag (#250114) was purchased from Zen Bioscience (Chengdu, China). The antiUNC5B (#13851), antiintegrinβ1 (#9699), antiPCNA (#2586)), antiCDK4 (#12790), anticyclin E2 (#4132), antiBax (#5023), anticleaved caspase-3 (#9661), antiBcl-2 (#15071), antiβ-actin (#4970), antiFAK (#3285), antiphospho-FAK (#3283), antiAkt (#9272), antiphospho-Akt (#9271), antiPI3K (#4292), antiphospho-PI3K (#13857), antiP50 (#3035), antiphospho-P50 (#4806), antiP65 (#3034), and antiphospho-P65 (#3039) antibodies used in western blotting assay were purchased from Cell Signaling Technology Inc. (Danvers, USA). All antibodies were diluted to the optimum concentration, according to the manufacturer’s instructions (1:1,000 dilution). Goat antirabbit immunoglobulin G (IgG) (horseradish peroxidase [HRP]) secondary peroxidase (#511203,1:5,000 dilution) and goat antimouse IgG (HRP) secondary antibody (#511103, 1:5,000 dilution) were purchased from Zen Biosciences (Chengdu, China).

### Elisa

Two milliliters of peripheral blood was collected from each patient using a vacuum tube without an anticoagulant. The blood samples were kept at room temperature for 2 h and then centrifuged at 1,000×g for 20 min at 4°C. The serum supernatant was separated and subjected to ELISA.

The human netrin-1 ELISA kit (E-EL-H2338c) was purchased from Elabscience (Wuhan, China), and ELISA was performed according to the manufacturer’s protocol at about 26–28°C. The netrin-1 levels were measured after establishing a standard curve for netrin-1 concentration. Each sample was analyzed five times per experiment.

### Cell culture

The K562 and THP-1 cells were purchased from American Type Culture Collection (Manassas, VA, USA) and identified using short tandem repeat profiling. The mycoplasma tests for these two cell lines yielded negative results. Both cell types were cultured in the Roswell Park Memorial Institute (RPMI) 1640 medium supplemented with 10% fetal bovine serum (FBS; Gibco, Thermo Fisher Scientific, Inc., Waltham, MA, USA) containing 100 U/mL penicillin and 100 U/mL streptomycin. The cells were incubated in a humidified atmosphere with 5% CO_2_ at 37°C, and the medium was changed every other day until the cells reached confluency. The cells were cryopreserved in liquid nitrogen in RPMI 1640 medium containing 10% dimethyl sulfoxide and 20% FBS.

### Cell counting Kit-8 cell growth detection

The number of cells seeded in the 96-well plate was counted using the CountStar software. Growth curves were obtained using CCK-8 (HY-K0301, MCE China, Shanghai, China) daily for 3 days (0 h, 24 h, 48 h, and 72 h). The number of cells was determined by measuring the absorbance at 450 nm using a microplate reader (Synergy 4, BioTek, Vermont, USA). Each group was measured five times per experiment, and each experiment was repeated three times in parallel.

### Flow cytometry analysis of cell apoptosis

The percentage of apoptotic cells was measured using an Annexin V-APC/7-AAD double-staining cell apoptosis detection kit (KGA1026; KeyGen Biotech Inc., Nanjing, China). The cells were stained following the manufacturer’s protocol using the supplied kit, and the stained cells were counted using flow cytometry. Each group was analyzed in parallel three times. The apoptotic rate was defined as the percentage of early and late apoptotic cells among all cells in the sample (Q2 and Q3, respectively).

### Western blotting and co-IP

Equal amounts of protein (30 μg/lane) were separated using sodium dodecyl sulfate-polyacrylamide gel electrophoresis (PG110, PG112, and PG113; Epizyme Inc., Shanghai, China) and then transferred to polyvinylidene difluoride membranes (Millipore, Darmstadt, Germany). After blocking with 5% defatted milk powder, the membranes were incubated with primary antibodies. After washing and incubation with the appropriate HRP-conjugated secondary antibody, the immune complexes were visualized using ultrasensitive ECL Western HRP Substrate (#17047, Zen Biosciences Inc.) and detected using a chemiluminescence reagent (Bio-Rad Laboratories, Inc., Hercules, CA, USA). All experiments were repeated thrice in parallel to confirm the accuracy of the results. β-actin was used as an internal control.

The cells were lysed with immunoprecipitation buffer (#88804; Pierce, Thermo Fisher Scientific, Inc.) in the presence of a proteinase inhibitor cocktail. The lysate was clarified by centrifugation at 13,000 × g for 10 min, and the protein concentration was measured using a bicinchoninic acid (BCA) protein assay (#P0010, Beyotime, Beijing, China). The protein (500 μg) was incubated with the indicated antibodies or mouse IgG overnight at 4°C. After being captured with beads for 1 h at room temperature, the immune complex was eluted with elution or neutralization buffer and boiled in one-quarter volume of 5× protein loading buffer (LT101, Epizyme Inc.) prior to detection by western blotting.

### RNAi to the UNC5B

The constructed RNAi retroviral UNC5B virus (NM_001244889.1) was purchased from Hanbio Biotechnology (Shanghai, China). The UNC5B interference sequences are listed in Supplementary Table S1. An RNAi sequence targeting green fluorescent protein (GFP) mRNA was used to construct an empty control vector. Transfection of 293T cells, viral titer selection, and infection were performed according to the manufacturer’s instructions. Two-to-three days after infection, a medium with 1 μg/mL puromycin (MA0318, Meilunbio, Dalian, China) was used for selection of stably infected strain. These cells were continually cultured with medium containing 0.5 μg/mL puromycin for further experiment. Interference with UNC5B expression was confirmed through western blotting.

### RT-PCR and total protein extraction

Total RNA was extracted using the TRIzol reagent (Invitrogen, Thermo Fisher Inc.), according to the manufacturer’s protocol and reverse-transcribed into complementary deoxyribonucleic acid using the RT Master Mix Kit for quantitative PCR (HY-K0510, MCE China). The SYBR Green RT-PCR Kit (HY-K0523, MCE, China) was used for amplification reactions using a Bio-Rad real-time PCR detection system according to the manufacturer’s protocol. Fluorescence curves were analyzed using Bio-Rad Software. β-actin was used as a control. The primers used for quantitative RT-PCR are listed in Supplementary Table S2.

Total protein was extracted using the Total Protein Extraction Kit (BB3101; Bestbio, Nanjing, China), according to the manufacturer’s instructions. The protein concentration was determined through BCA protein assay; the product was added to a one-quarter volume of 5×loading buffer before boiling at 100°C for 10 min and then stored at − 20°C for further experiments.

### Statistical analysis

All statistical analyses were performed using the Statistical Package for the Social Sciences version 26.0 (IBM Corp., Armonk, NY, USA). Data were expressed as mean±standard deviation for data with a Gaussian distribution and median (minimum-maximum) for data with non-Gaussian distribution. The serum netrin-1 levels between the two groups were analyzed using the Mann – Whitney U test, and the differences among multiple groups were analyzed using the Kruskal – Wallis test. The differences between groups of experimental data followed a Gaussian distribution and were analyzed using one- or two-way analysis of variance, followed by the Bonferroni post hoc test, which compared the differences between groups of experimental data. *P* < .05. *P < .05, **P < .01, ***P < .001, and ****P < .0001 were considered significant.

## Supplementary Material

Supplemental Material

## Data Availability

The authors confirm that the data supporting the findings of this study are available within the article and its supplementary materials.

## Health and safety statement

All mandatory laboratory health and safety procedures have been complied during the course of the experiment.
