# Supplementary material for: Netrin-1 inducing antiapoptotic effect of acute myeloid leukemia cells in a concentration-dependent manner through the Unc-5 netrin receptor B-focal adhesion kinase axis
Source: Cancer Biol Ther. 2023 Apr 10;24(1):2200705. doi: 10.1080/15384047.2023.2200705 (PMC10088980; doi:10.1080/15384047.2023.2200705)
Supplement: Supplemental Material [file KCBT_A_2200705_SM5959.docx]

**Supplementary Table S1: Interfering RNA sequence for UNC5B**

| UNC5B | Forward strand | 5′-gatccGCCACACAGATCTACTTCAATTCAAGAGATTGAAGTAGATCTGTGTGGTTTTTTg-----3′ |
| --- | --- | --- |
|  | Reverse strand | 5′-aattcAAAAAACCACACAGATCTACTTCAATCTCTTGAATTGAAGTAGATCTGTGTGGCg-----3′ |

**Supplementary Table S2: Primers for real-time quantitative PCR**

| ITGβ1  (NM_002211.4) | Forward strand | 5′-TTGACCACAGTTGTTACGG-3′ |
| --- | --- | --- |
|  | Reverse strand | 5′-ATGACAGAAGGGAGTTTGC-3′ |
| NEO1  (NM_001172623.1) | Forward strand | 5′-AAAGAGGGGAAACCTAAGACCA-3′ |
|  | Reverse strand | 5′-CCCACAACAGGCTCAATAACC-3′ |
| DCC  (NM_005215.3) | Forward strand | 5′-AGCCGATTTGTCCGTCTCA-3′ |
|  | Reverse strand | 5′-TGTTGTATTCAATGCTCGTTCC-3′ |
| UNC5A  (NM_133369.2) | Forward strand | 5′-TGGCTGACTCGTCCATTCTC-3′ |
|  | Reverse strand | 5′-GTGGTGGTGGTGGTGCTGA-3′ |
| UNC5B  (NM_170744.4) | Forward strand | 5′-GTCCTTGAACATTAGCGGTTT-3′ |
|  | Reverse strand | 5′-GACACGCCTGTAGCACTGAA-3′ |
| UNC5C  (NM_003728.3) | Forward strand | 5′-GCGGACTGGGACTGGGATA-3′ |
|  | Reverse strand | 5′-GTGGCAGAGGCTCAGGTGG-3′ |
| UNC5D  (NM_080872.2) | Forward strand | 5′-CAAGGACTGGCAGATGTTAGC-3′ |
|  | Reverse strand | 5′-TTCAAAATGACAGCAGATGGG-3′ |
